# Supplementary material for: Longitudinal Plant Health Monitoring via High-Resolution Mass Spectrometry Screening Workflows: Application to a Fertilizer Mediated Tomato Growth Experiment
Source: Molecules. 2023 Sep 22;28(19):6771. doi: 10.3390/molecules28196771 (PMC10574498; doi:10.3390/molecules28196771)
Supplement: Supplementary file 1 [file molecules-28-06771-s001.zip › molecules-2575435-supplementary.pdf]

molecules

MDPI

Supplementary Material

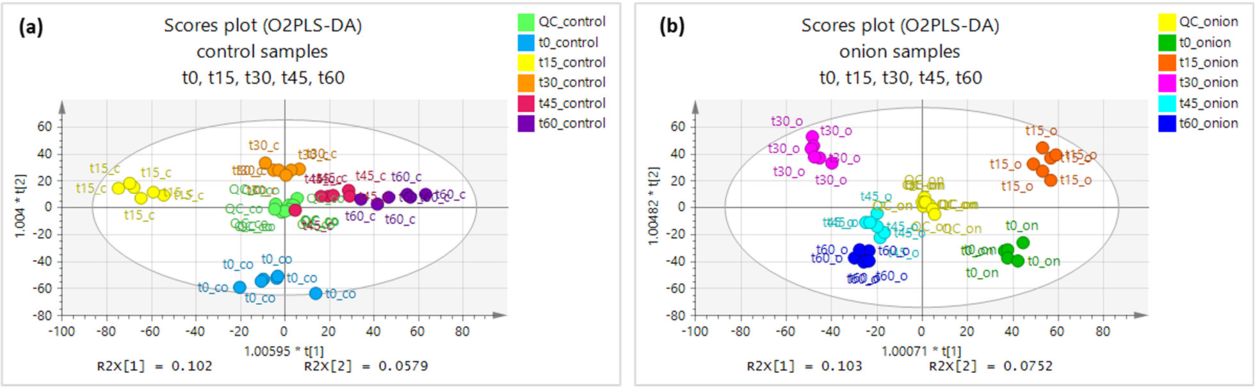

Figure S1: Score plot O2PLS-DA of (a) control samples and (b) onion samples at five time points (t0, t15, t30, t45, t60) with their corresponding QC samples using UV scaling in positive ionization mode.

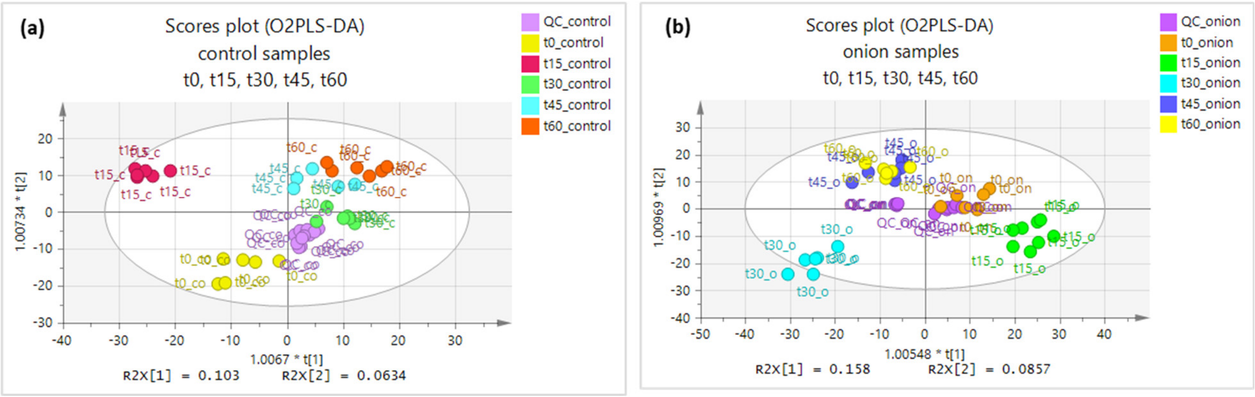

Figure S2: Score plot O2PLS-DA of (a) control samples and (b) onion samples at five timepoints (t0, t15, t30, t45, t60) with their corresponding QC samples using UV scaling in negative ionization mode.

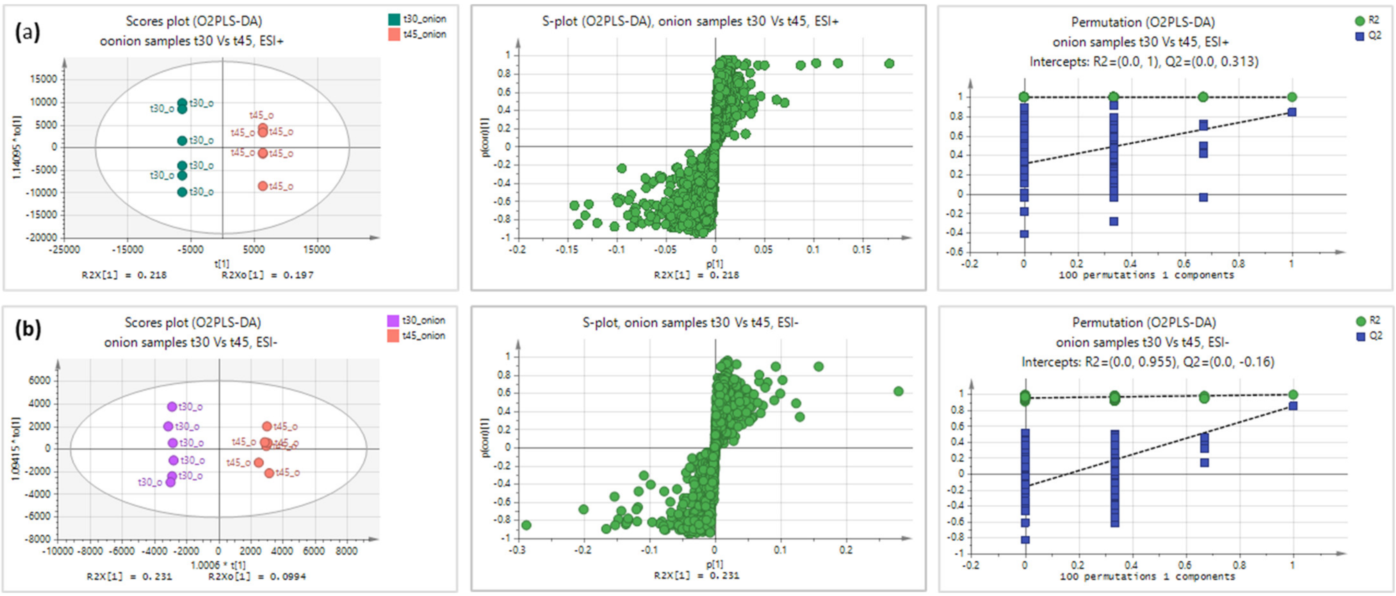

Figure S3: Score plot O2PLS-DA discrimination using pareto scaling, S-Plot, and permutation testing for onion samples at t30 vs. t45 in (a) positive ionization mode and (b) negative ionization mode.

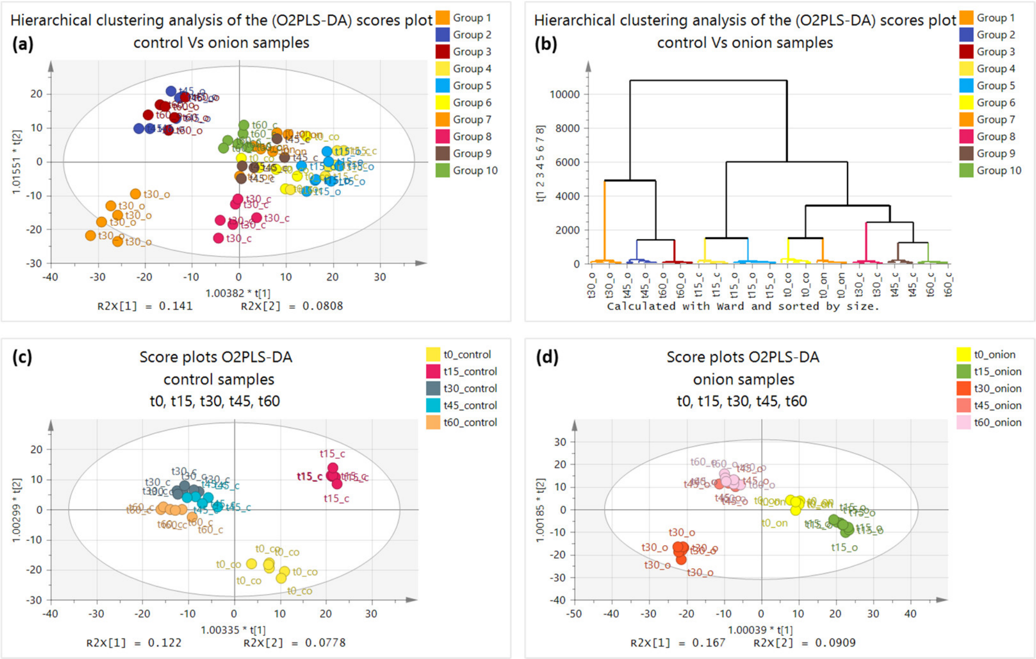

Figure S4: Plots of O2PLS-DA and hierarchical clustering analysis: (a) score plots of control and onion samples (b) hierarchical clustering analysis O2PLS-DA of control and onion samples; score plot O2PLS-DA discrimination between (c) control samples and (d) onion samples at five timepoints (t0, t15, t30, t45, t60) using UV scaling in negative ionization mode.

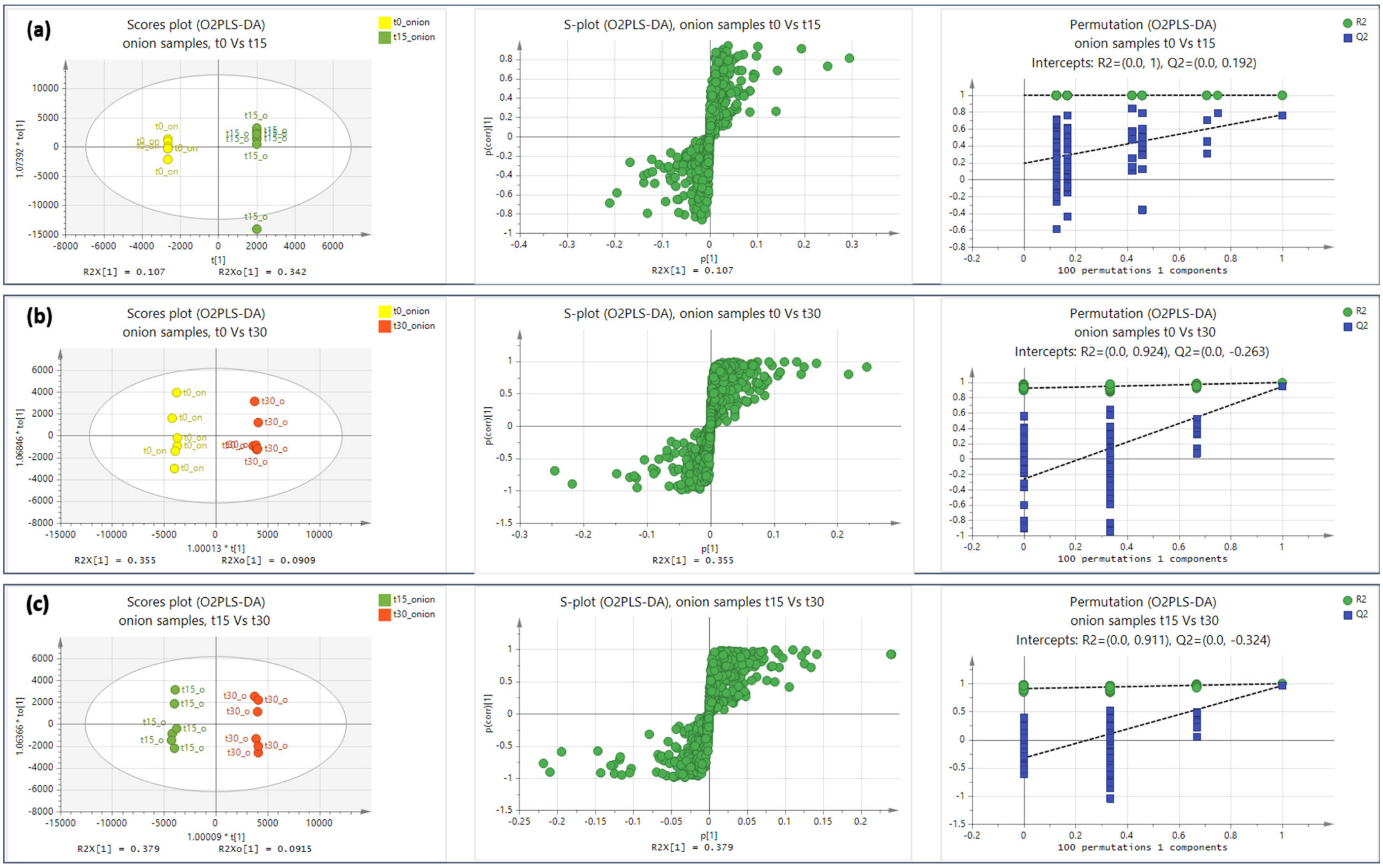

Figure S5: Score plot O2PLS-DA discrimination using pareto scaling, S-Plot, and permutation testing for onion samples at (a) t0 vs. t15, (b) t0 vs. t30, and (c) t15 vs. t30 in negative ionization mode.

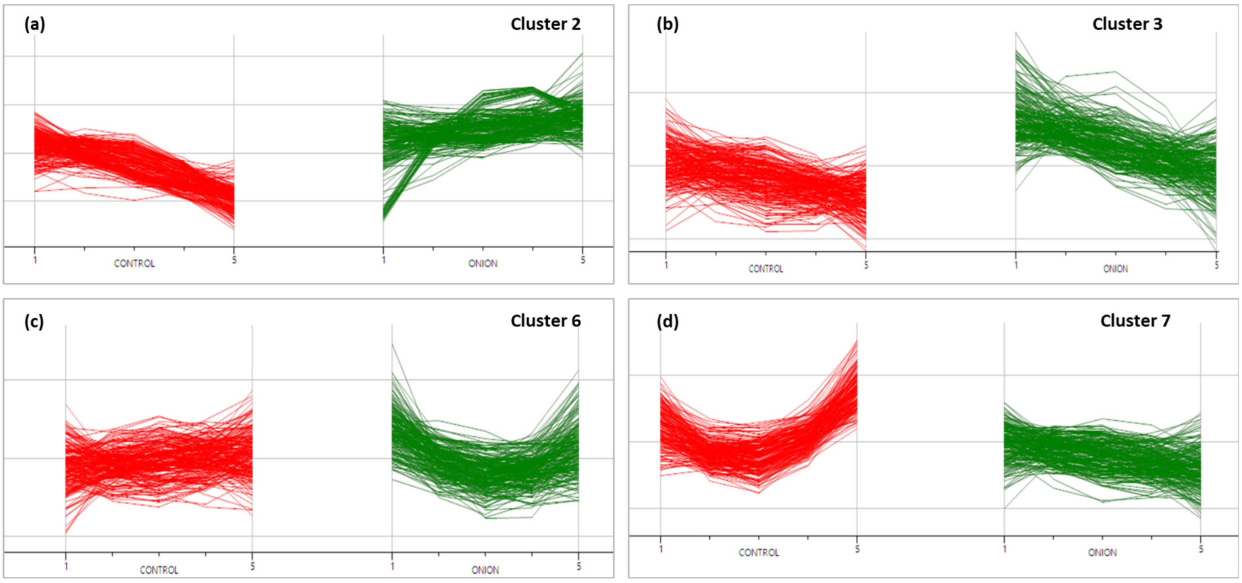

Figure S6: Examples of compound clustering between control (in red) and onion samples (in green) in positive ionization mode using MetaboClust: (a) Cluster 2, (b) Cluster 3, (c) Cluster 6, and (d) Cluster 7.

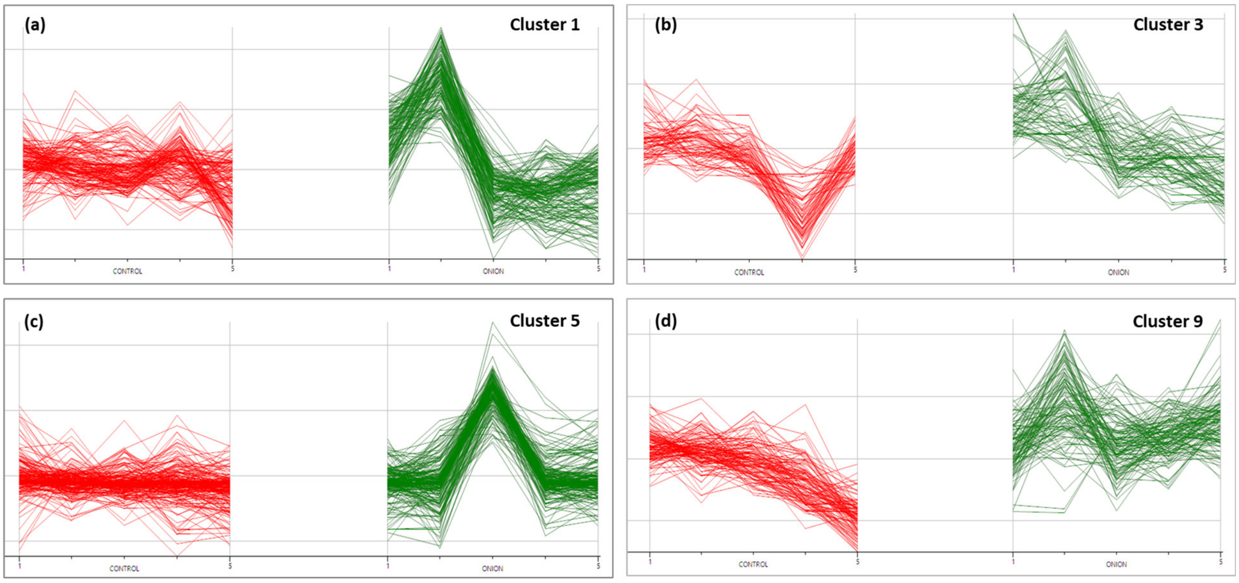

Figure S7: Examples of compound clustering between control (in red) and onion samples (in green) using MetaboClust: (a) Cluster 1, (b) Cluster 3, (c) Cluster 5, and (d) Cluster 9 in negative ionization mode.

Table S1. Performance characteristics of each classification model in **positive** ionization mode

Investigated samples	R^2 (Y)	Q^2	*p*-values	Permutation values		Fisher's probabil-ities
				R^2	Q^2	
Controls and Onions: t0, t15, t30, t45, t60	0.532	0.391	1.97×10^{-5}	0.349	-0.207	0
Controls: t0, t15, t30, t45, t60	0.973	0.572	1.46×10^{-4}	0.909	-0.179	8.8×10^{-17}
Onions: t0, t15, t30, t45, t60	0.985	0.671	1.28×10^{-6}	0.925	-0.231	8.8×10^{-17}
t0 onion, t15 onion	1	0.897	1.54×10^{-1}	1	0.37	0.0022
t0 onion, t30 onion	0.997	0.948	5.52×10^{-4}	0.909	-0.285	0.0022
t15 onion, t30 onion	0.999	0.938	1.48×10^{-3}	0.991	-0.135	0.0011

Table S2. Performance characteristics of each classification model in negative ionization mode

Investigated samples	R^2(Y)	Q^2	*p*-values	Permutation values		Fisher's probabil-ities
				R^2	Q^2	
Controls and Onions: t0, t15, t30, t45, t60	0.791	0.458	2.27×10^{-3}	0.514	-0.319	0
Controls: t0, t15, t30, t45, t60	0.971	0.655	5.27×10^{-4}	0.877	-0.239	6.1×10^{-16}
Onions: t0, t15, t30, t45, t60	0.973	0.755	4.70×10^{-10}	0.889	-0.265	9.9×10^{-19}
t0 onion, t15 onion	1	0.767	3.79×10^{-1}	1	0.192	0.00033
t0 onion, t30 onion	0.998	0.946	1.60×10^{-4}	0.924	-0.263	0.0011
t15 onion, t30 onion	0.999	0.964	3.82×10^{-5}	0.911	-0.324	0.0023
